# Case Report: Anesthesia for cloacal prolapse fixation surgery in an Argentine black and white tegu (*Salvator merianae*)

**DOI:** 10.3389/fvets.2026.1770284

**Published:** 2026-03-23

**Authors:** Ying Liu, Ludi Zhu, Min Yan, Ruilong Wang, Jia Liu, Mingchao Zhao, Jianhua Xiao, Chongwei Hu

**Affiliations:** 1 Department of Veterinary Surgery, Northeast Agricultural University, Harbin, Heilongjiang, China; 2 Department of Animal Science, Fujian Agricultural University, Fuzhou, Fujian, China; 3 Mondy Animal Hospital, Fuzhou, Fujian, China

**Keywords:** anesthesia, cloacal prolapse, lizards, mechanical ventilation, reptiles

## Abstract

Cloacal prolapse is a common surgical condition in captive lizards and frequently requires general anesthesia. Anesthetic management in reptiles is challenging because of their ectothermic physiology, intermittent breathing patterns, and high sensitivity to anesthetic-induced respiratory depression. This report describes the anesthetic management of a juvenile Argentine black and white tegu (*Salvator merianae*) under surgical reduction of cloacal prolapse. Anesthesia was induced using a multimodal protocol including butorphanol, tiletamine-zolazepam, dexmedetomidine, and maintained with isoflurane. Marked respiratory depression occurred after induction, necessitating controlled mechanical ventilation throughout the procedure. Ventilatory support and active thermal management were applied to maintain physiologic stability. This case support the importance of mechanical ventilation, temperature control, and multimodal analgesia in anesthetic management of large lizard species.

## 1 Introduction

In current clinical cases involving exotic companion animals, cloacal prolapse is a relatively common condition in reptiles, and anesthesia is often an essential component during surgical reduction (1). However, anesthetic management in reptiles-particularly in lizards, is different from that in dogs, cats, and other mammals. This difference is primarily attributable to the absence of a diaphragm in reptiles, pulmonary ventilation therefore relies mainly on active movements of the intercostal muscles and axial skeletal musculature to alter thoracic cavity volume (2, 3). Under general anesthesia, lizards frequently exhibit marked respiratory depression or even apnea (4, 5). Consequently, controlled ventilation using a mechanical ventilator is often required during anesthetic management in lizards to maintain effective alveolar ventilation and gas exchange. Furthermore, although previous studies have clearly demonstrated that reptiles possess the capacity for pain perception and are capable of exhibiting behavioral and pharmacological responses to noxious stimuli (6–8), the subtle and highly species-specific of pain expression in reptiles makes anesthetic monitoring and perioperative analgesic management particularly challenging (7). Against this background, the present report describes the anesthetic management and perioperative analgesic strategy employed in a cloacal prolapse reduction surgery performed in an Argentine black and white tegu (*Salvator merianae*).

## 2 Case description

**Figure 1:**
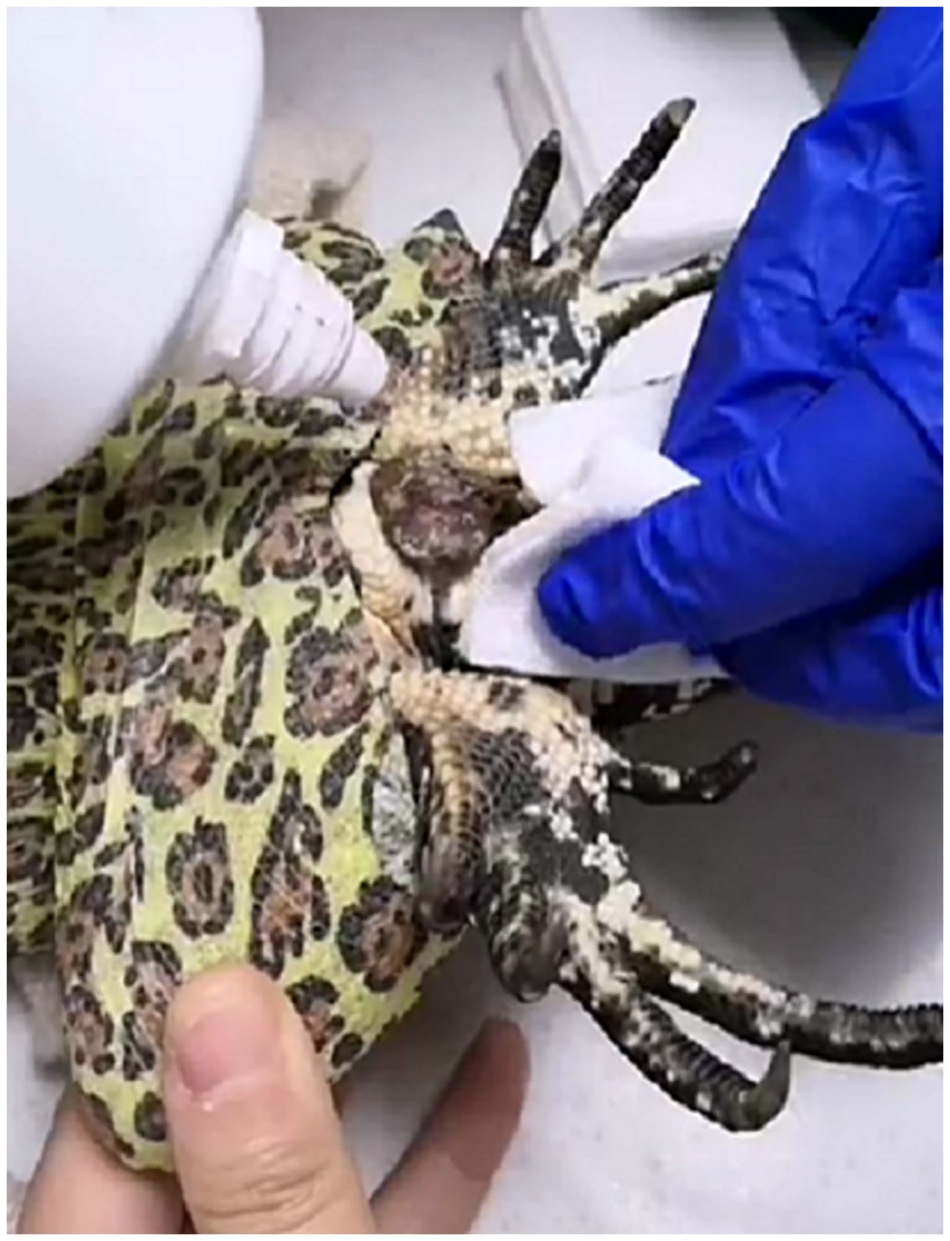
A mass protruding from the cloacal region.

For A 1.5 year old, male, 1.68 kg, client-owned Argentine black and white tegu with a history of diarrhea and a mass protruding from the cloacal region (Figure 1). The owner reported that recently this lizard showed decreased appetite, while urination and defecation remained normal. Husbandry included UVA + UVB lighting, with an ambient temperature of 35 °C in the basking area and 28–30 °C in the shaded area, and was fed mice and vegetables every 3–4 days, with vitamin and calcium supplementation provided with each meal and electrolyte supplementation every 1–2 weeks. It had fallen from a height of approximately 1 m one week ago, with bleeding from the right nostril at that time. On initial presentation, physical examination revealed a bright, alert lizard with moist, pink oral mucous membranes, a body condition score of 4/9, and adequate hydration. No abnormalities were detected on palpation of the body surface. Respiratory rate (fR) was 16 breaths min^−1^ and heart rate was 60 beats min^−1^. Hematologic evaluation using a blood smear revealed an increased heterophil count (5.06 × 10^3^/μL; reference 2.2 ± 0.45 × 10^3^/μL), increased lymphocyte count (10.1 × 10^3^/μL; reference 7.5 ± 0.58 × 10^3^/μL), and a packed cell volume of 24% (reference 25 ± 2.6%). Based on a comprehensive evaluation of the lizard’s clinical signs, physical examination findings, and hematological test results, a diagnosis of cloacal prolapse was established, and surgical reduction of the cloaca under anesthesia was deemed necessary.

**Figure 2:**
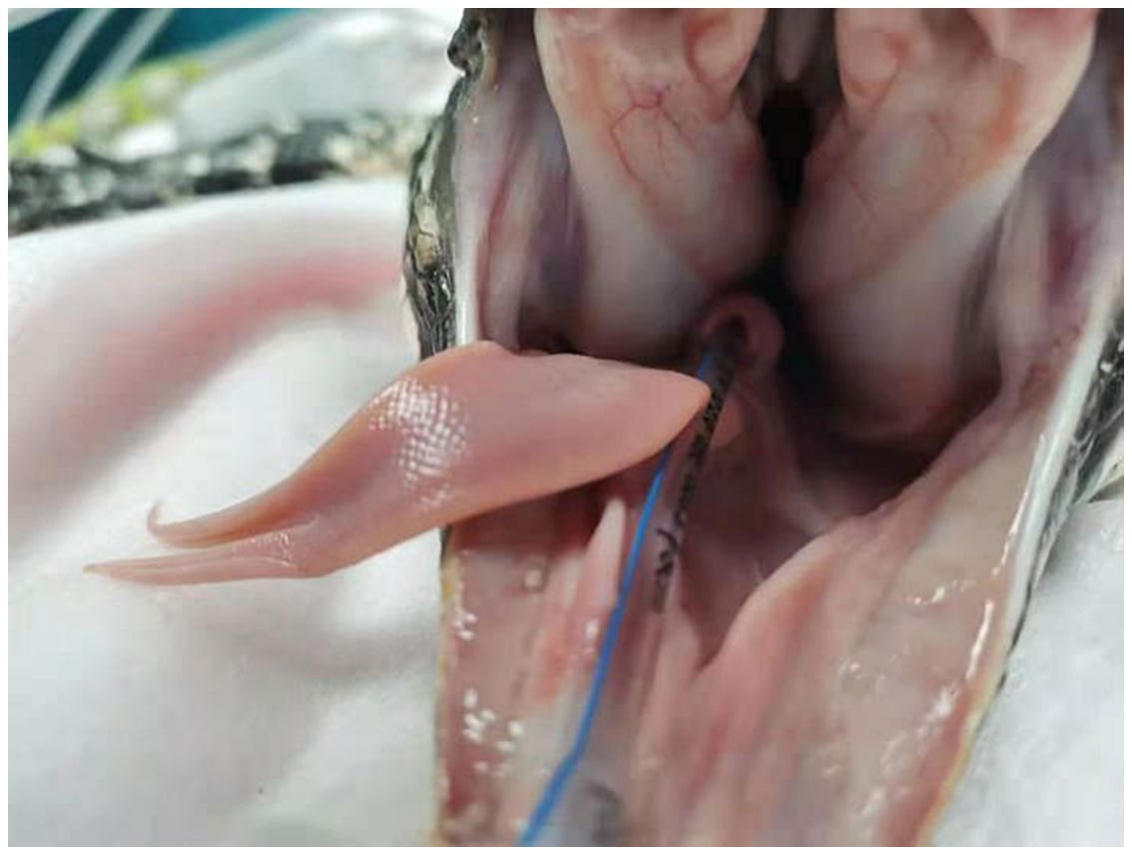
The lizard was intubated with a 2.5 mm uncuffed endotracheal tube.

**Figure 3:**
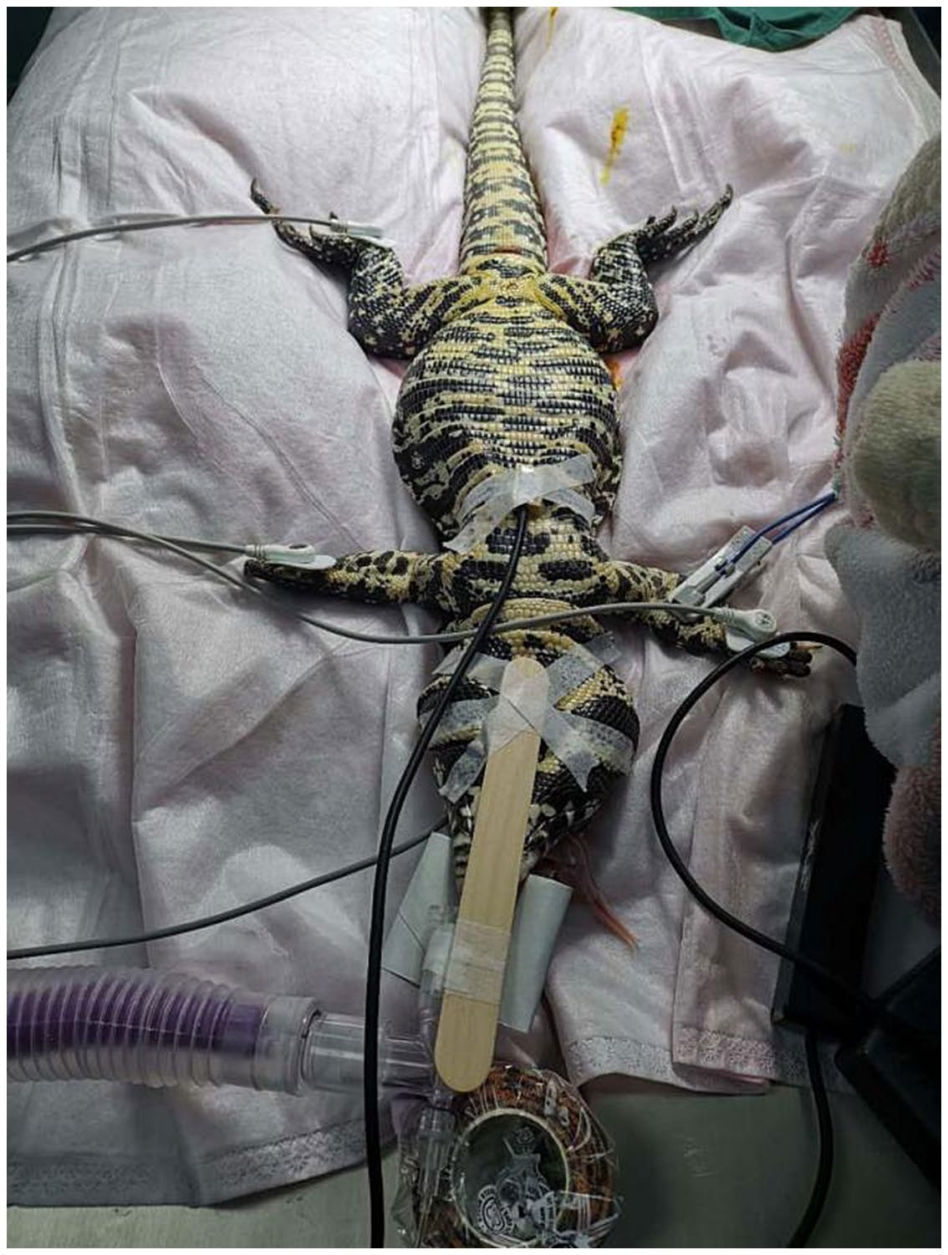
Electrocardiography, peripheral oxygen saturation, and end-tidal carbon dioxide were monitored, and body temperature was maintained with Bair Hugger.

The lizard was fasted for 24 h prior to surgery. Both the ambient temperature and the forced-air warming blanket (Bair Hugger forced-air warming blanket, model/3M, St. Paul, MN, USA) were set at 30 °C. Preoperatively, enrofloxacin was administered subcutaneously at a dose of 5 mg/kg (diluted; Baytril®, Bayer Healthcare Co., Ltd., Beijing, China). Prior to anesthesia, the lizard underwent a thermoneutral acclimation period, during which external light sources were turned off. Sedation was induced by intramuscular injection into the forelimb muscles of a mixture containing butorphanol (0.5 mg/kg; Yibeile® veterinary butorphanol injection, Haimen Huiju Pharmaceutical Co., Ltd., Haimen, China), tiletamine/zolazepam (1 mg/kg; Zoletil®, Virbac, Carros, France), and dexmedetomidine (0.05 mg/kg; Zoetis Inc., Shanghai, China). Supplemental oxygen was provided using a gas mixture of 40% oxygen and 60% air for 10–15 min until loss of muscle tone and relaxation of the jaw were observed. As the spontaneous respiratory rate decreased, the lizard gradually became apneic, and was rapidly intubated using a 2.5 mm uncuffed endotracheal tube (Well Lead Medical Co., Ltd., Guangzhou, China) (Figure 2). Anesthesia was maintained by anesthesia workstation (WATO 35, Mindray Bio-Medical Electronics Co., Ltd., Shenzhen, China) with oxygen and isoflurane (Beiyining®, Hainan Lingkang Pharmaceutical Co., Ltd., Hainan, China). The patient was immediately connected to a multiparameter monitor (ePM 12M Vet, Mindray Bio-Medical Electronics Co., Ltd., Shenzhen, China) for continuous monitoring of electrocardiography, oxygen saturation and end-tidal parameters. Body temperature was maintained throughout the procedure using the Bair Hugger (Figure 3). A catheter was placed in the right cephalic vein (22-gauge, 1 inch, 25 mm; KRUUSE, Langeskov, Denmark), and lactated Ringer’s solution (100 mL; Otsuka, Shanghai, China) was administered intravenously at a rate of 5 mL/kg/h. Anesthetic parameters and events were recorded using a standardized anesthesia record form recommended by the Veterinary Anesthesia Association.

**Figure 4:**
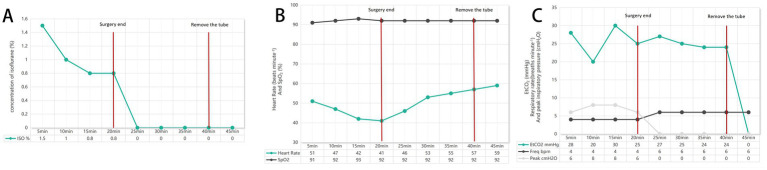
**(A)** Isoflurane concentration. **(B)** Heart Rate and SpO_2_. **(C)** EtCO_2_ Respiratory rate and peak inspiratory pressure.

The isoflurane concentration varied from 0.8 to 1.5% throughout surgery (Figure 4A). The heart rate ranged from 41 to 59 beats min^−1^, and the peripheral capillary oxygen saturation (SpO_2_) remained between 91 and 93% (Figure 4B). Throughout anesthesia, the lizard was mechanically ventilated using pressure-controlled ventilation (PCV) to maintain gas exchange, with the following settings: peak inspiratory pressure varied 6–8cmH_2_O, and fR is 4 breaths min^−1^ to maintain end-tidal carbon dioxide between 20 and 30 mmHg (4.7–5.9 kPa) (Figure 4C).

A percutaneous cloacopexy was performed. A lubricated cotton swab was inserted into the cloaca and positioned adjacent to the lateral abdominal wall. Topical anesthesia with lidocaine hydrochloride 2 mg/kg (Hefeng Pharmaceutical Co., Ltd., Shanghai, China) was applied at the intended suture sites. 2-0 PDS sutures (Johnson & Johnson Medical, Shanghai, China) were passed percutaneously through the abdominal wall and cloacal wall over the swab. The swab was then removed through the vent, the sutures were detached from the swab tip, the suture loops were drawn back into the cloacal lumen, and the cloaca was anchored to the lateral abdominal body wall.

The surgical procedure lasted 20 min. At the end of the procedure, isoflurane was discontinued, and residual anesthetic gases were flushed from the breathing circuit. The endotracheal tube was maintained, and the animal continued to receive 40% oxygen and 60% air. At this time, the lizard had not regained spontaneous respiration, so mechanical ventilation was continued, and the ventilator settings were adjusted accordingly: airway pressure 6–8 cmH_2_O, respiratory fR is 6 breaths min^−1^, while recovery from anesthesia was awaited. Five minutes after surgery, the lizard resumed spontaneous respiration, and mechanical ventilation was discontinued. Twenty minutes later, mandibular tone and hindlimb muscle tone gradually returned. Endotracheal extubation was performed when tongue retraction was observed. Continuous physiological monitoring was maintained until the animal was removed from the operating table. Once the lizard was able to lift its head and ambulate, it was transferred to the ward for postoperative observation.

The lizard was discharged after recovering from anesthesia and regaining normal ambulation, and was returned to its owner 6 h after admission. Meloxicam (Boehringer Ingelheim, Germany) was administered orally at a dose of 0.5 mg/kg once every 24 h for 3 days. A follow up consultation 4 weeks after surgery confirmed a return to normal behavior and function.

## 3 Discussion

### 3.1 The relationship between temperature, cardiac shunting, and oxygen in reptiles

Temperature is critical in reptile anesthesia because their body temperature is largely determined by the ambient environment (9). Temperature fluctuations beyond the preferred optimal temperature range (POTR) can significantly affect the onset of anesthesia, the duration of drug metabolism, and recovery time, making anesthetic depth more difficult to control (10). In this case, the routine husbandry temperature was 28–35 °C, therefore, during anesthesia we set both the ambient temperature and the air-warming blanket to 30 °C, which helped stabilize ventilation and circulation, maintain good control of anesthetic depth, and improve the lizard’s recovery quality.

Compared with mammals, a key difference in reptilian cardiopulmonary physiology is the presence of an incompletely divided ventricle, which permits dynamic intracardiac shunting (11). The direction and magnitude of intracardiac shunting (right-to-left or left-to-right) are actively regulated through changes in pulmonary and systemic vascular resistance (12). In this lizard, because arterial blood gas analysis was not performed, so it’s unclear whether any shunting occurred. However, because the monitored inhalant anesthetic concentration and the depth of anesthesia showed generally consistent trends, we infer that this lizard essentially did not develop intracardiac shunting, or that only a small amount of intracardiac shunting may have been present without affecting the anesthetic depth.

In these animals, the fraction of inspired oxygen also modulates respiratory drive and interacts with intracardiac shunting (13, 14). The use of a high inspired oxygen concentration may exacerbate respiratory depression by blunting the hypoxic ventilatory drive, resulting in a marked reduction in minute ventilation (14, 15). Therefore, prolonged use of 100% oxygen should be avoided during anesthesia in lizards; instead, room air or gas mixtures with a lower oxygen concentration are recommended (14, 15). In this case, we maintained anesthesia using a gas mixture of 40% oxygen and 60% air to minimize the risk of reduced minute ventilation associated with high inspired oxygen concentrations.

In reptiles, during warming or periods of increased metabolic demand, pulmonary perfusion increases and left-to-right shunting predominates, thereby enhancing pulmonary gas exchange. In contrast, during cooling, apnea, or reduced metabolic demand, right-to-left shunting becomes more pronounced, reducing pulmonary blood flow and conserving energy (16, 17). Meanwhile, a high oxygen concentration can reduce ventilation in reptiles, and combined with a persistent cardiac right-to-left shunt, it may paradoxically impair effective oxygen delivery despite an increase in arterial partial pressure of oxygen. Therefore, to manage potential intracardiac shunting, we maintained anesthesia in this lizard using an appropriate ambient temperature and a lower inspired oxygen concentration.

Humans and other mammals possess a fully divided four-chambered heart, intracardiac shunting is not present under normal physiology and instead reflects abnormal communications (e.g., congenital shunt lesions) (18). Ventilation and perfusion are therefore tightly coupled, and increases in inspired oxygen concentration typically improve arterial oxygenation without inducing significant ventilatory suppression (19). Although hyperoxia may blunt peripheral chemoreceptor activity to some extent, its effects on minute ventilation are modest and do not produce the profound respiratory depression observed in reptiles (20). These physiological differences underscore the importance of appropriate thermal management, cautious oxygen supplementation, and a thorough understanding of cardiac shunting mechanisms when anesthetizing reptiles. Failure to consider the interactions among temperature, oxygen availability, ventilation, and intracardiac shunting may result in significant hypoventilation, altered gas exchange, and delayed anesthetic recovery.

### 3.2 Controlled mechanical ventilation in lizards following loss of spontaneous breathing under anesthesia

In reptiles, particularly lizards, spontaneous respiration is readily depressed or may cease entirely during general anesthesia. Consequently, the initiation of mechanical ventilation following the establishment of inhalational anesthesia is considered a routine and essential practice (4). Previous studies have shown that in lizards experiencing prolonged anesthetic-induced apnea, spontaneous breathing often fails to resume even when arterial carbon dioxide levels rise, indicating that a “wait-and-see” approach to restoring spontaneous respiration is inappropriate for this species.

In this lizard, once muscle tone was lost and jaw relaxation was achieved, we promptly performed endotracheal intubation and maintained ventilation with a mechanical ventilator. We chose pressure-controlled ventilation (PCV) because an uncuffed endotracheal tube was used, making it impossible to estimate tidal volume or calculate the volume of leaked gas. Therefore, compared with volume-controlled ventilation (VCV), PCV is better suited to reptilian respiratory physiology and is less likely to result in hypoventilation or overinflation. Current clinical and conference literature generally recommends the use of low-frequency IPPV during lizard anesthesia, with ventilation rates of approximately 4 breaths min^−1^ and sufficient expiratory time to accommodate their predominantly passive expiratory mechanics (4). After surgery and discontinuation of isoflurane, this lizard did not immediately resume spontaneous ventilation, therefore, mechanical ventilation needed to be continued. During this period, spontaneous respiratory efforts should be closely monitored and/or the ventilator switched to an assisted mode to prevent ventilator dyssynchrony, as well as hypercapnia and cardiovascular depression resulting from premature cessation of ventilation.

The application of mechanical ventilation in anesthetized lizards aims not only to improve oxygenation but, more importantly, to prevent hypercapnia and maintain anesthetic stability (21, 22). This functions more as a life-supporting replacement for spontaneous respiration than as a merely adjunctive intervention (23).

### 3.3 Local anesthesia and analgesia in lizards

Topical anesthesia can reduce the overall dose of general anesthetic agents required (24). In this case, we used lidocaine hydrochloride for topical anesthesia. According to our anesthesia record, at a stable isoflurane concentration, we did not observe any marked increase in heart rate during suturing, indicating that topical anesthesia at a dose of 2 mg/kg provided effective local analgesia.

In lizards, lidocaine diffuses locally into the tissues surrounding peripheral nerve endings and produces analgesia by inhibiting sodium ion influx in local nerve cells (5, 25). It has a relatively rapid onset, and its pharmacokinetic characteristics appear similar to those observed in dogs, cats, rabbits, and other small mammals (5). In our experience, when a lizard’s stratum corneum and scale structure are relatively thick, the effectiveness of topical anesthetics may be reduced, and pronounced inadequate analgesia may occur. Conversely, in areas with local skin damage or missing scales, rapid absorption of local anesthetics may occur; therefore, the animal’s specific skin condition should be carefully taken into account when selecting the dose.

Local anesthesia is more often regarded as an adjunctive technique in lizards, rather than a primary anesthetic modality (26). Similarly, for other superficial surgical procedures in lizards, local infiltration anesthesia may be applied at the surgical site to effectively reduce the requirements for additional analgesic and general anesthetic agents.

In conclusion, the anesthetic protocol described in this analysis of the Argentine black and white tegu may serve as a practical reference for routine surgical anesthesia in this species. Notably, the described ventilatory management can be implemented for most lizards that lose spontaneous respiration when a surgical plane of anesthesia is achieved. These findings contribute to the refinement of anesthetic protocols and provide valuable guidance for analgesia and ventilatory management in diverse reptile species.

## Data Availability

The original contributions presented in the study are included in the article/Supplementary material, further inquiries can be directed to the corresponding authors.

## Supplementary material

SUPPLEMENTARY FIGURE 1 Anesthesia was recorded using a standardized anesthetic recordform recommended by the Association of Veterinary Anaesthetists. (AVA).
