# Supplementary figures and images for: Case Report: Anesthesia for cloacal prolapse fixation surgery in an Argentine black and white tegu (Salvator merianae)
Source: Front Vet Sci. 2026 Mar 23;13:1770284. doi: 10.3389/fvets.2026.1770284 (PMC13051652; doi:10.3389/fvets.2026.1770284)

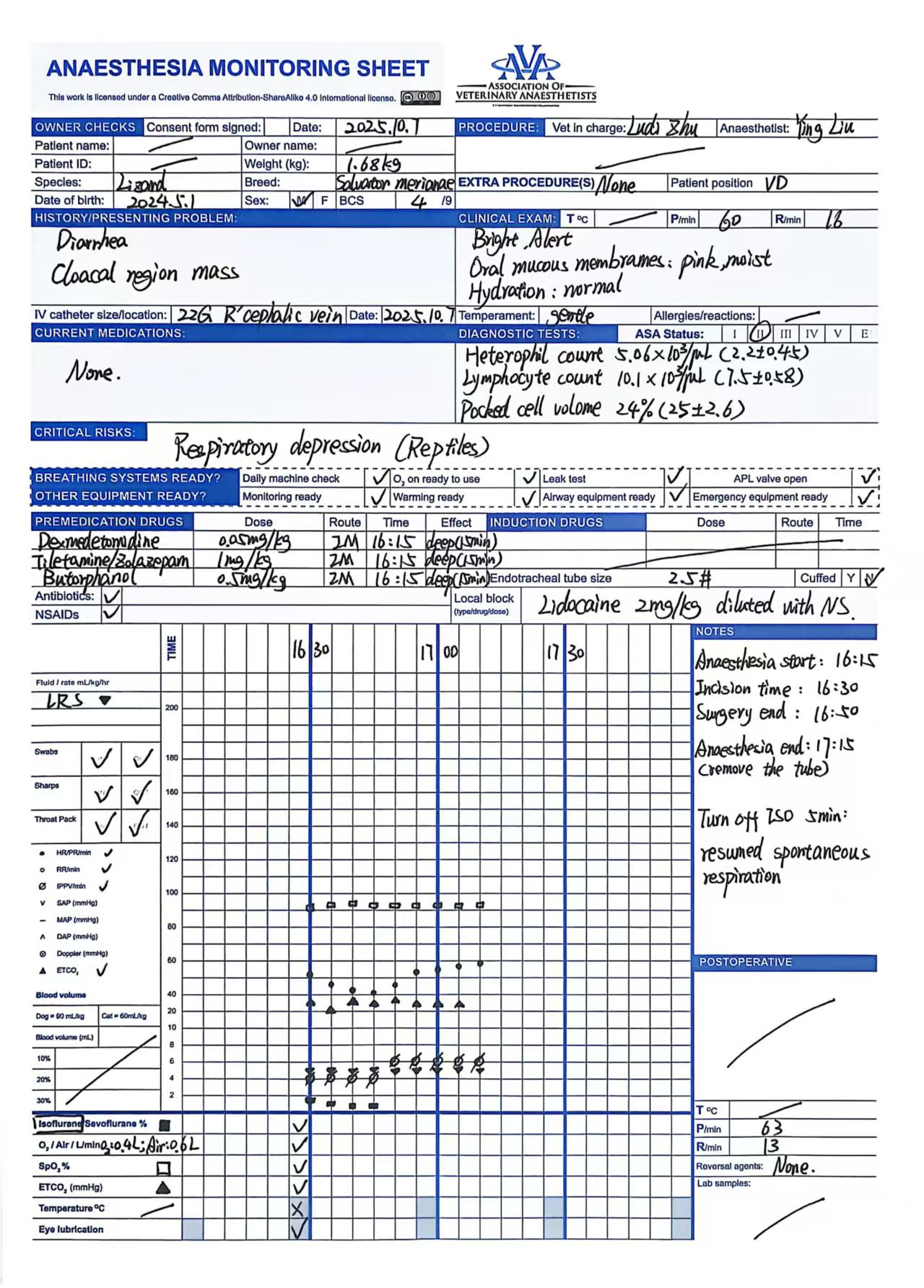

Supplement: SUPPLEMENTARY FIGURE 1 — Anesthesia was recorded using a standardized anesthetic recordform recommended by the Association of Veterinary Anaesthetists. (AVA). [file Image_1.TIF]
